# Electrical resistivity tomography (ERT) data for clay mineral mapping

**DOI:** 10.1016/j.dib.2020.105494

**Published:** 2020-04-08

**Authors:** Rungroj Arjwech, Pakawat Sriwangpon, Kittipong Somchat, Potpreecha Pondthai, Mark Everett

**Affiliations:** a Department of Geotechnology, Faculty of Technology, Khon Kaen University, Khon Kaen 40002, Thailand; b Department of Geology and Geophysics, Texas A&M University, College Station, Texas 77843-3115, USA

**Keywords:** 2D electrical resistivity tomography, Clay mineral, Kaolinite, Ranong province

## Abstract

In order to identify potential zones for clay mineral (kaolinite) in Ranong province, Thailand, ten 2D ERT survey lines were deployed across potential site. The data were collected using the IRIS Syscal Pro Plus multi-electrode imaging system with internal switchbox and an array of 48 steel electrodes. The Dipole-Dipole configuration was utilized with electrode separations of 10 m. The Res2DInv software was used to process the data. Images for the resistivity survey are presented as 2D cross section of resistivity profile. The data were interpreted by comparing with geology of the area and based on the available lithological borehole information.

Specifications table

**Table:** 

Subject	Earth and Planetary Sciences
Specific subject area	Geophysics-2D Electrical resistivity tomography
Type of data	Figure and Binary (.bin) files
How data were acquired	The 2 ERT data were acquired using Syscal Pro Resistivity Meter with 48 electrodes by IRIS Instruments.
Data format	Raw.
Parameters for data collection	The Dipole-Dipole array was utilized with electrode separations of 10 m. Model cells with widths of half unit spacing was used for inversion process.
Description of data collection	Ten ERT survey lines were selected according to the available site accessibility across potential site around intrusive rock hill.
Data source location	The site is located at Ban Haad Som Pan, Haad Som Pan Subdistrict, Muang District, Ranong Province. UTM Grid 465700-466300E and 1099200-1099700N Series L7018 Map Sheet 4728 I
Data accessibility	With the article

## Value of the data

The method to get these data is cost-effective, rapid, non-destructive and generates relevant, spatially continuous subsurface information.
The datasets can be used for characterisation of the subsurface profile which is useful for mining.
The data are extremely applicable in subsurface investigation such as mineral exploration and groundwater and can be integrated with other geophysical data sets.
The data are used for integrated mine decisions that can reduce cost of further drilling to locate mineral zone.
Integration of dataset from boreholes and ERT survey lines is used for mine planning.

## 1 Data description

**Fig. 1:**
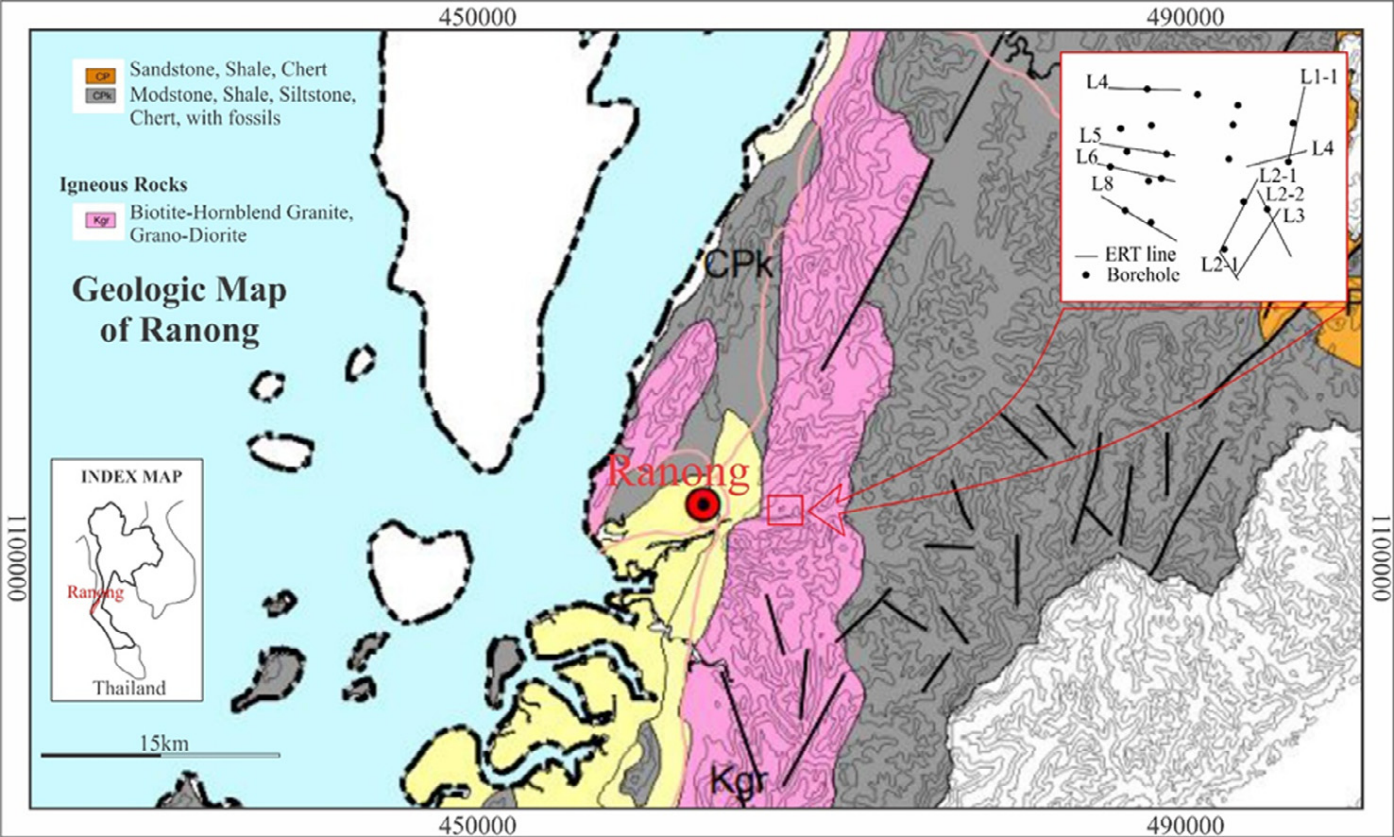
Geologic map of Ranong Province, Thailand.

**Fig. 2:**
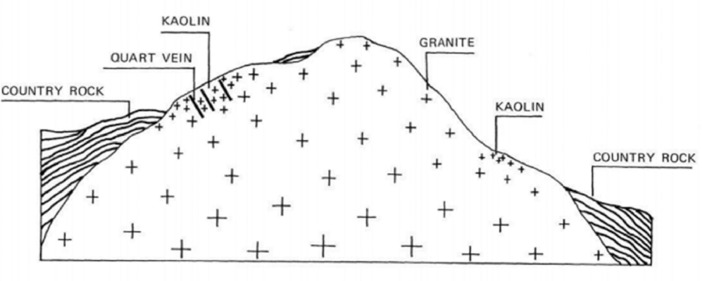
Ideal section of granite intrusion and kaolin deposit in Haad Som Pan area [1].

**Fig. 3:**
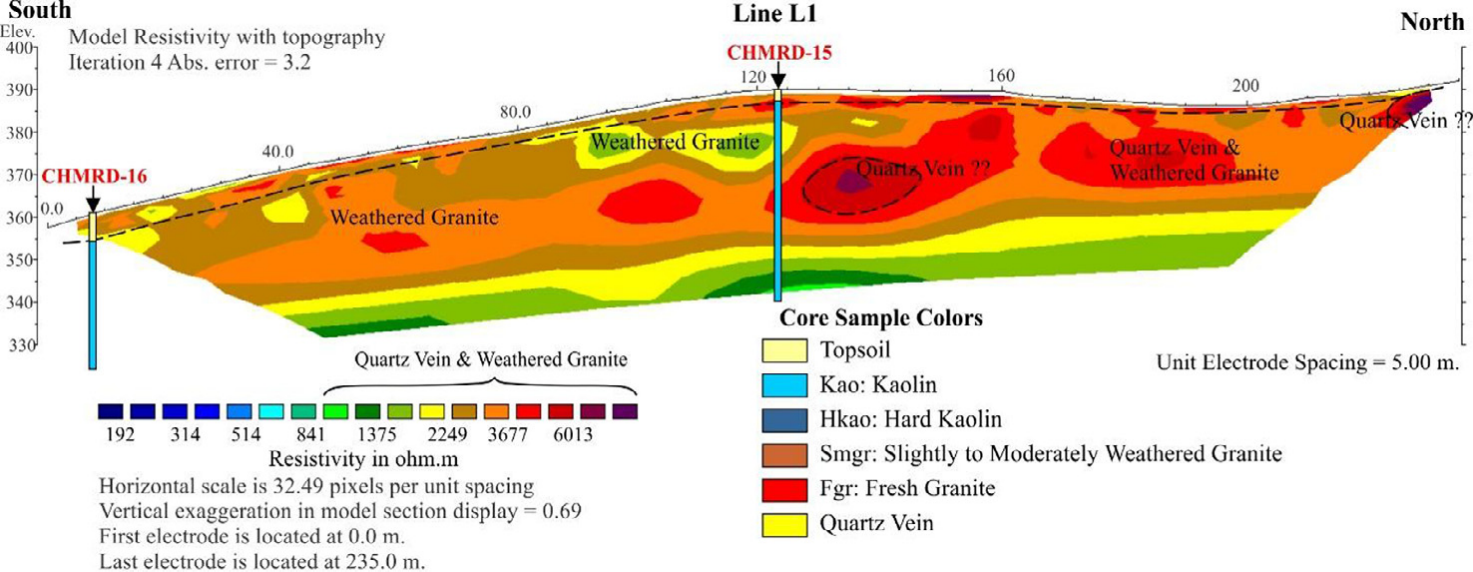
The inversion image of Line L1.

**Fig. 4:**
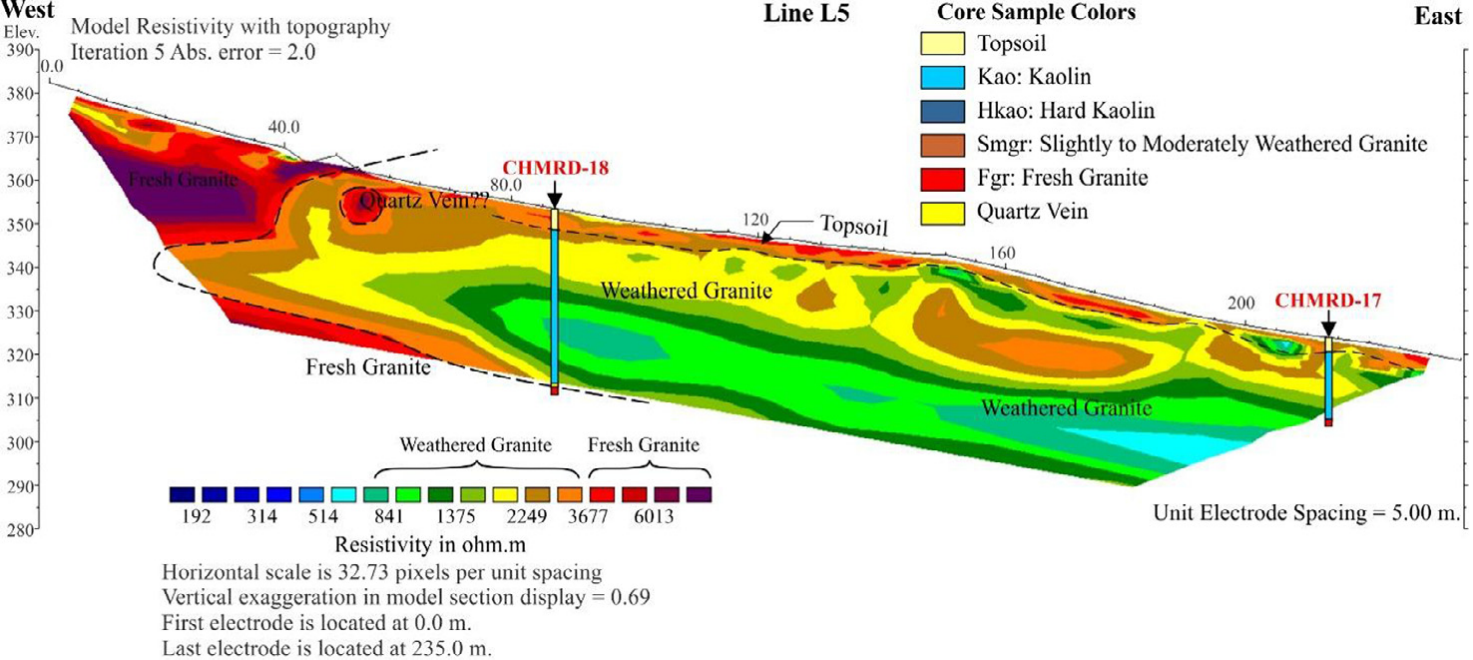
The inversion image of Line L5.

**Fig. 5:**
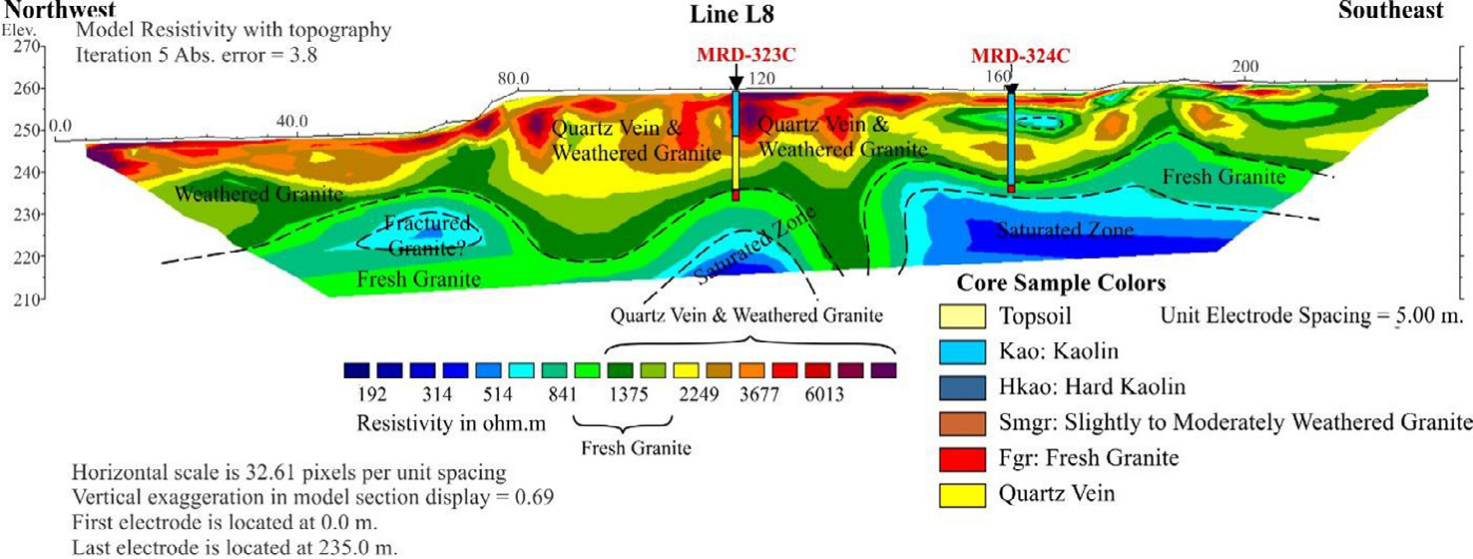
The inversion image of Line L8.

The 2D ERT survey was conducted over terrain (Figs. 1 and 3) where clay mineral, weathering of granite rock (Fig. 2), was partly noticed on ground surface. The data measured in the field were recorded in .bin file (supplementary data), mainly consisting of apparent resistivity, electric current, potential, standard deviation, and location of electrodes. Elevations along the survey lines were measured and added later to resistivity data via Prosys II software that later were converted to .dat file. Res2Dinv inversion software directly reads and processes the .dat format. The inversion images show high resolution of 2D cross section with locations of boreholes, short description of lithology and interpretation shown in Figs. 4–6. The attached files (Appendices A) consist of ten lines of 2D ERT data.

## 2 Experimental design, materials, and methods

The site lies within granitic rocks that were formed as a batholith as a part of the high mountain range in Ranong Province, Thailand (Fig. 1). The clay deposits that belongs to a consequence of weathering of granite rock from the pneumatolysis of feldspar, it also can be seen that some of the kaolin clays are associated with the quartz veins (Fig. 2) [1].

The ERT data were collected along ten lines (Fig. 1 inset) using the Syscal Pro Plus multi-electrode imaging system with internal switchbox and an array of 48 steel electrodes. The dipole–dipole array configuration was used with electrode spacing 10 m and the length is between 200–235 m. Elevation data were also measured along the resistivity profiles.

The Res2DInv software was used to process the resistivity data. The software uses a forward and inverse modelling procedure to create a synthetic data set based on measured apparent resistivity. This is an iterative process; a root-mean-square (RMS) error is calculated for each new iteration. Noisy data points are progressively removed over the course of several iterations until the RMS error is reduced to an acceptable level. The profiles terrains were corrected using elevation data collected along the ERT lines. The Res2DInv software incorporates the elevation data into the inverse modelling procedures.

Images for the resistivity survey are presented as cross section of resistivity profile. The data were interpreted by comparing with geology of the area and matching with values of electrical resistivity of earth materials. Rock boundaries indicated on the resistivity profiles are certainly based on the available lithological borehole information.

The images obtained, which are based on the 2D inversion of the field data. The color bar indicates the range of electrical resistivity values in unit of ohm-meters (Ωm). The color scale is logarithmic and consistent with contour intervals. Cool colors (i.e. blue) represent areas of low resistivity values. Warm colors (i.e. red) represent areas of high resistivity values [2,3]. The weathered granite is interpreted from the resistivity values between 1000–3000 Ωm (Figs. 3–5).

## Appendix Supplementary materials

Image, application 1

## References

[1] Uttamasil L. (1969). An investigation of Ranong clay from Thailand, the faculty of the graduate division. Georgia Inst Technol.

[2] Sarntima T., Arjwech R., Everett M. (2019). Geophysical mapping of shallow rock salt at Borabue, Northeast Thailand. Near Surface Geophys.

[3] Arjwech R., Everett M. (2019). Electrical Resistivity tomography at construction sites with implications for building foundation design, Northeast Thailand. J Environ Eng Geophys.

